# Supplementary material for: Elucidating the Racemization Mechanism of Aliphatic and Aromatic Amino Acids by In Silico Tools
Source: Int J Mol Sci. 2023 Jul 25;24(15):11877. doi: 10.3390/ijms241511877 (PMC10418343; doi:10.3390/ijms241511877)
Supplement: Supplementary file 1 [file ijms-24-11877-s001.zip › ijms-2504301-supplementary.pdf]

In silico study of the racemization mechanism of aliphatic and aromatic amino acids

Supplementary Material

Index of Figures

Figure S1: Hammett σ plot using W_2 for calculations of kinetic constants. 2
Figure S2: Hammett σ plot using W_3 for calculations of kinetic constants. 2
Figure S3: Hammett σ plot using W_{23} for calculations of kinetic constants. 3
Figure S4: IRC breaking of C_1-H_5 bond profile for racemization of aromatic compounds. 3
Figure S5: IRC breaking of C_1-H_5 bond profile for racemization of aliphatic compounds. 4
Figure S6: Bond length difference from reactant to transition state. H_5-O_6 formation length diminishes and C_1-H_5 cleavage length increases. 4
Figure S7: Bond length difference from transition state to product. H_5-O_6 formation length diminishes and C_1-H_5 cleavage length increases. 5
Figure S8: IRC C_1-H_5-O_6 bond angle profile for racemization of aromatic compounds. 5
Figure S9: Bond length comparison between the C_1-H_5 bond and H_5-O_6 across IRC calculations. 6
Figure S10: IRC dipole profile for racemization of aromatic compounds. 7
Figure S11: IRC dipole profile for racemization of aliphatic compounds. 7
Figure S12: Aromatic amino acids structure in basic media. 8

Index of Tables

Table S1: Square correlation coefficient R^2 and slope m of linear regression between NBO charges of reactant (R), transition state (TS), and intermediate (I) and experimental energy of activation. 6

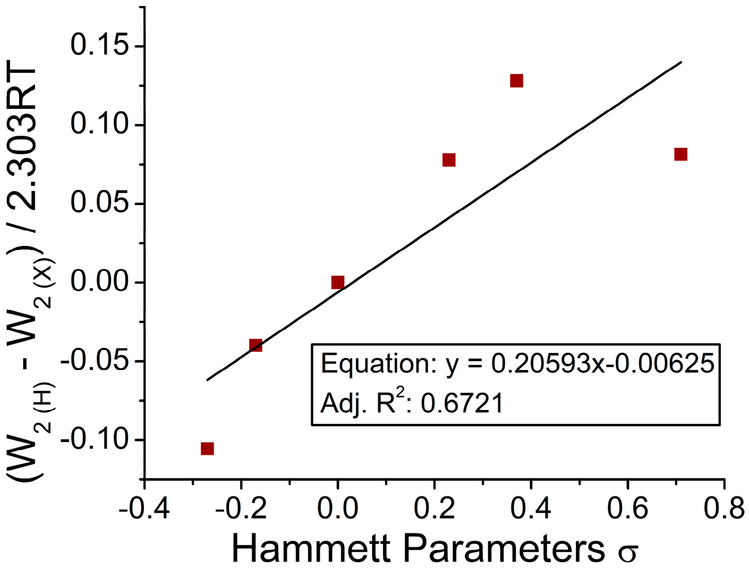

Figure S1: Hammett σ plot using W_2 for calculations of kinetic constants.

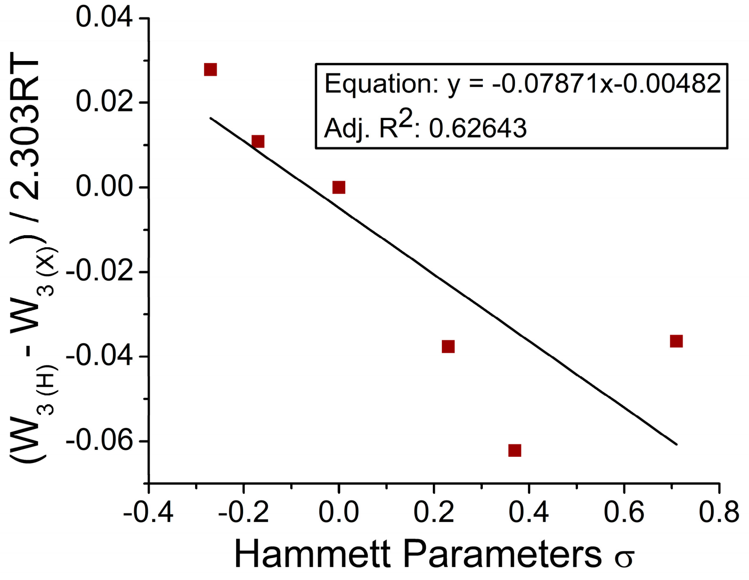

Figure S2: Hammett σ plot using W_3 for calculations of kinetic constants.

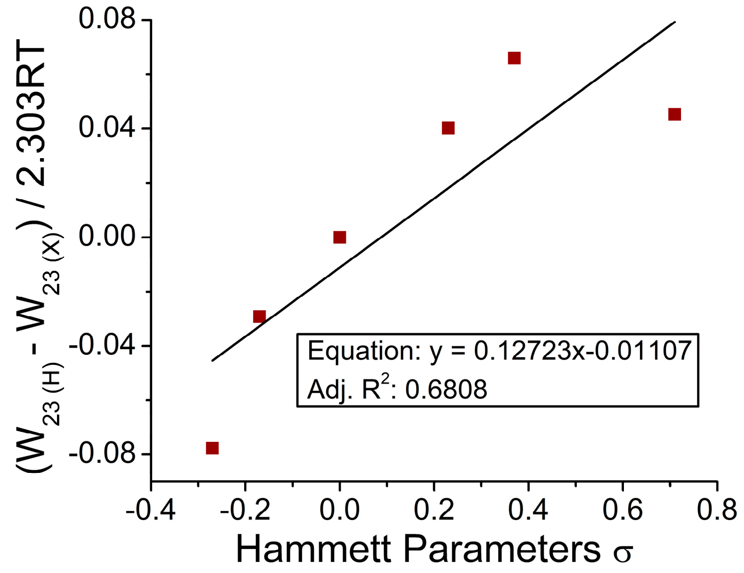

Figure S3: Hammett σ plot using W_{23} for calculations of kinetic constants.

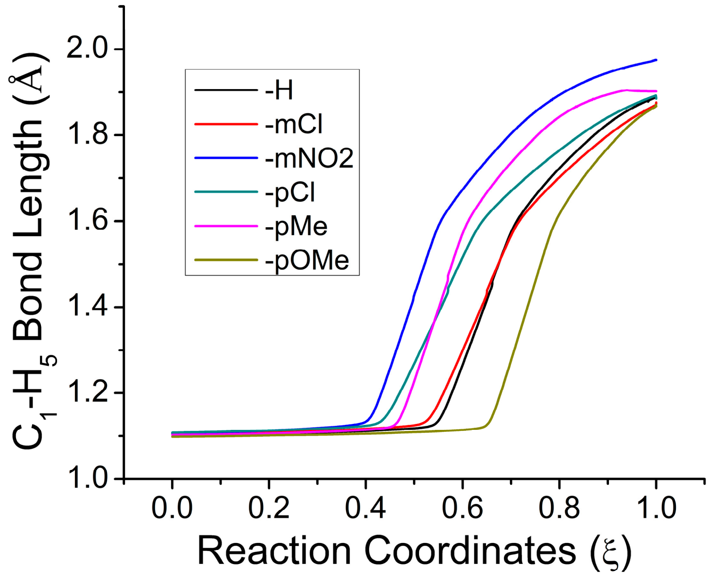

Figure S4: IRC breaking of C_1-H_5 bond profile for racemization of aromatic compounds.

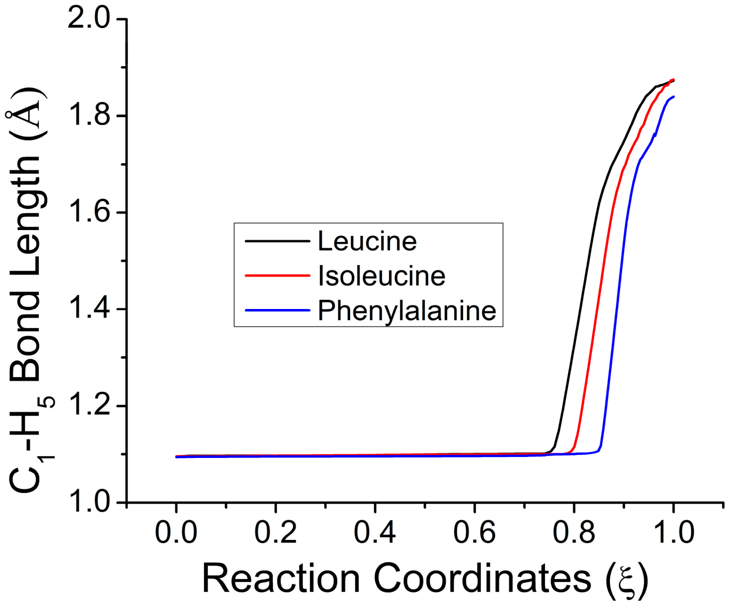

Figure S5: IRC breaking of C_1-H_5 bond profile for racemization of aliphatic compounds.

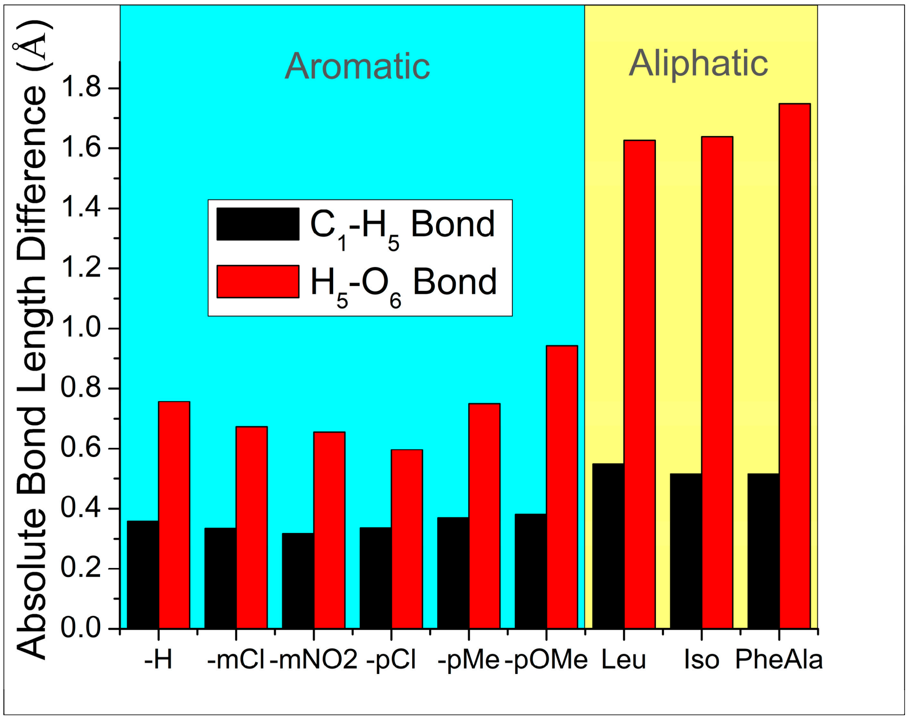

Figure S6: Bond length difference from reactant to transition state. H_5-O_6 formation length diminishes and C_1-H_5 cleavage length increases.

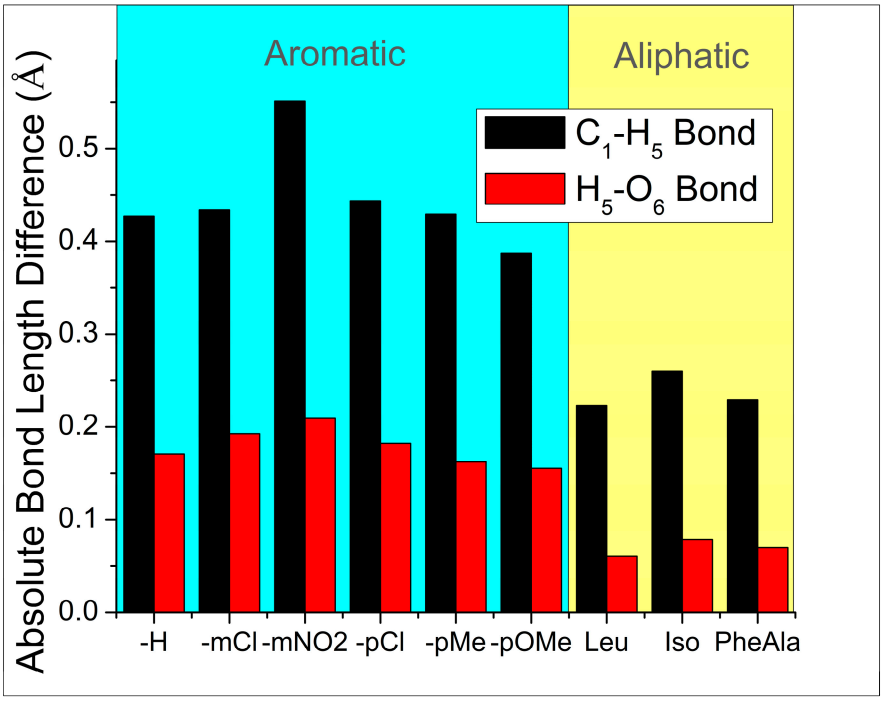

Figure S7: Bond length difference from transition state to product. H_5-O_6 formation length diminishes and C_1-H_5 cleavage length increases.

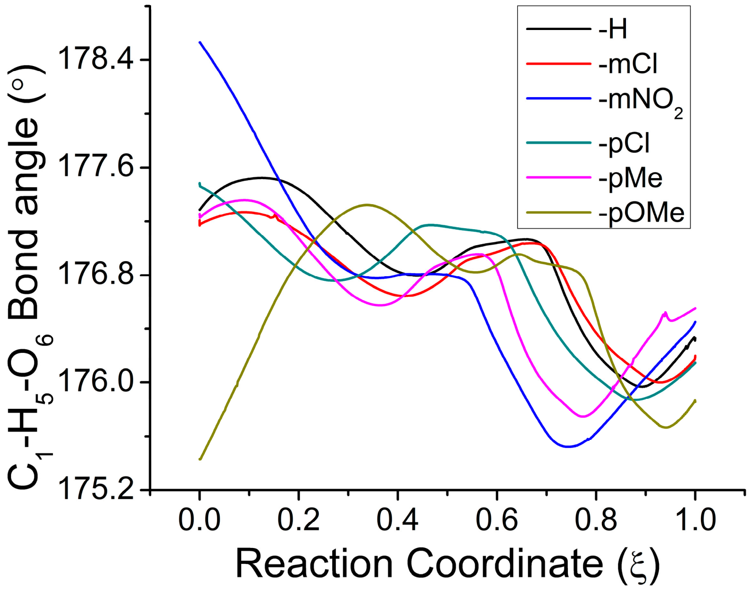

Figure S8: IRC C_1-H_5-O_6 bond angle profile for racemization of aromatic compounds.

Table S1: Square correlation coefficient R^2 and slope m of linear regression between NBO charges of reactant (R), transition state (TS), and intermediate (I) and experimental energy of activation.

Atom		R	TS	I	TS-R	I-TS	I-R
C_1	R^2	0.757[2]	0.178	0.013	0.721	< 0.001	0.602
	m	0.0110[3]	0.0004	0.0004	-0.0106	> -0.0001	-0.0107
C_2	R^2	0.026	0.906	0.982	0.951	< 0.001	0.983
	m	0.0002	-0.0078	-0.0078	-0.0080	>-0.0001	-0.0080
N_3	R^2	0.948	0.988	0.989	0.933	0.827	0.552
	m	-0.0025	-0.0039	-0.0032	-0.0014	0.0007	-0.0007
C_4	R^2	0.675	0.676	0.673	0.372	0.571	0.023
	m	-0.0434	-0.0446	-0.0431	-0.0012	0.0015	0.0003
H_5	R^2	0.865	0.907	0.385	0.454	0.833	0.184
	m	-0.0135	0.0044	-0.0008	0.0109	-0.0052	0.0058
O_6	R^2	0.753	0.804	0.759	0.701	0.799	0.014
	m	-0.0052	0.0064	-0.0032	0.0089	-0.0096	-0.0007

2. The highest correlation for each atom is highlighted in blue.
3. The highest absolute slope value for each atom is highlighted in purple.

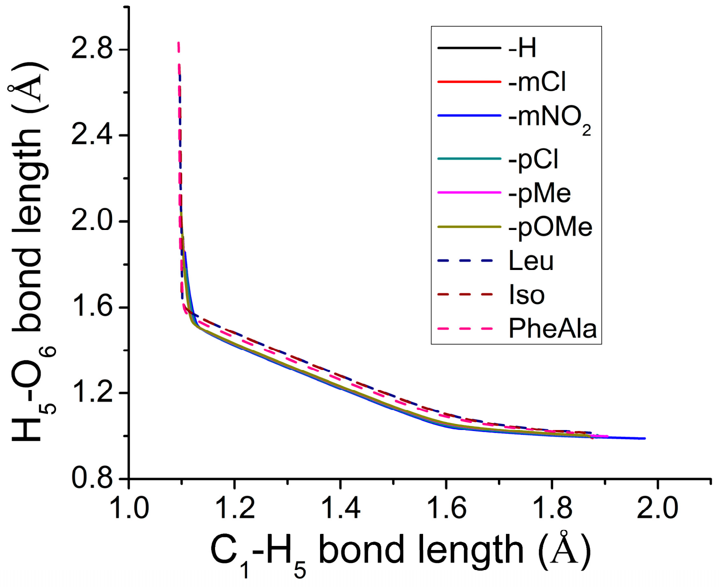

Figure S9: Bond length comparison between the C_1-H_5 bond and H_5-O_6 across IRC calculations.

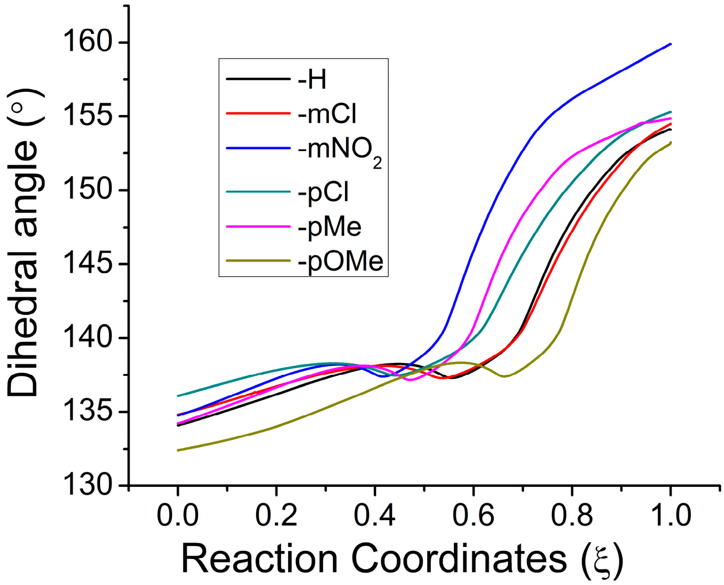

Figure S10: IRC dipole profile for racemization of aromatic compounds.

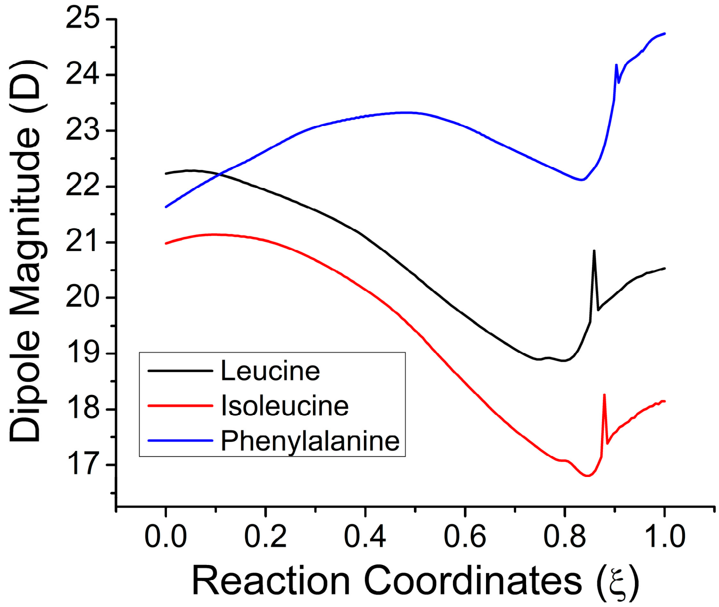

Figure S11: IRC dipole profile for racemization of aliphatic compounds.

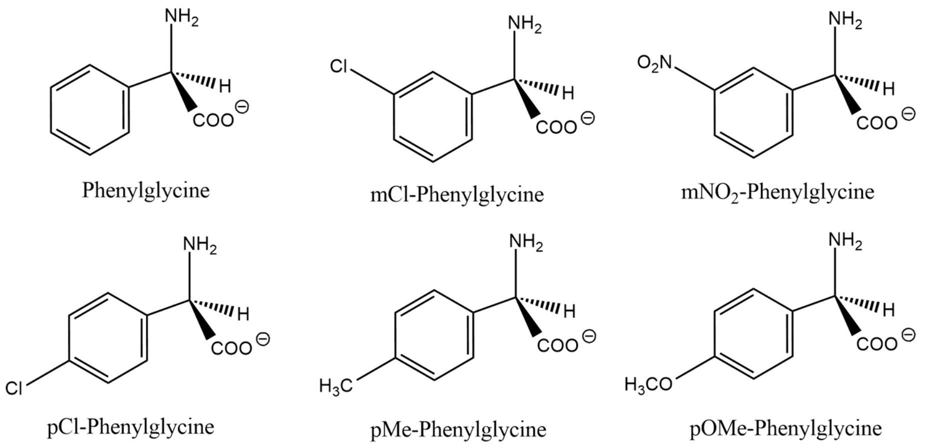

Figure S12: Aromatic amino acids structure in basic media.
